# Development of a clinical nomogram to assess the risk of cognitive impairment in community-dwelling middle-aged and older adults

**DOI:** 10.1186/s12883-026-04719-6

**Published:** 2026-02-11

**Authors:** Mengchen Wang, Yao Sun, Xiaoxiao Wang, Chun Liu, Tao Guo, Yu Huang, Frankliu Gao, Bensheng Qiu

**Affiliations:** 1 Department of Medical Imaging Technology, School of Medical Imaging, Bengbu Medical University, Bengbu, Anhui 233030 China; 2 School of Medical Information Engineering, Gannan Medical University, Ganzhou, Jiangxi China; 3 Department of Electronic Engineering and Information Science, Medical Imaging Center, University of Science and Technology of China, Hefei, Anhui 230026 China; 4 Michael G. Foster School of Business, University of Washington, Box 353200, Seattle, WA 98195-3200 USA

**Keywords:** Cognitive impairment, Nomogram, Risk factors, Middle-aged and elderly people

## Abstract

**Background:**

Cognitive impairment is a prevalent condition among middle-aged and older adults and often progresses to dementia, posing substantial clinical and societal burdens. Early assessment of high-risk individuals is essential for timely intervention and management. This study aimed to develop a practical nomogram for the assessment of cognitive impairment in community-dwelling elderly populations.

**Methodology:**

This cross-sectional study recruited 581 participants between October 23 and November 8, 2023, comprising 465 assigned to the training cohort and 116 to the validation cohort. Demographic information, medical history, lifestyle, and biochemical parameters were collected using structured questionnaires. Cognitive impairment was assessed via the Montreal Cognitive Assessment (MoCA). Independent features were identified using LASSO regression followed by binary logistic regression, and a nomogram was constructed based on these variables. Model performance was evaluated by discrimination, calibration, and clinical utility using Receiver Operating Characteristic (ROC) curves, calibration plots, the Hosmer–Lemeshow test, and Decision Curve Analysis (DCA).

**Results:**

Cognitive impairment prevalence was 38.5% in the training and 32.8% in the validation cohort. Six features—sex, age, systolic blood pressure, homocysteine, fruit consumption, and family history of stroke—were integrated into the nomogram. The model demonstrated good discrimination (AUC 0.816 in training cohort; 0.796 in validation cohort) with satisfactory calibration and clinical applicability.

**Conclusion:**

The proposed nomogram provides a reliable and convenient tool for the early risk assessment of cognitive impairment in middle-aged and older adults, facilitating targeted prevention and personalized management in clinical and community settings. Its implementation may assist healthcare professionals in identifying high-risk individuals and mitigating progression toward dementia.

**Supplementary Information:**

The online version contains supplementary material available at 10.1186/s12883-026-04719-6.

## Introduction

Cognitive impairment, characterized as a deficit in one or more cognitive domains, varies in severity and stems from diverse etiologies [1]. Alzheimer's Disease (AD) represents its most severe manifestation [2]. This condition predominantly affects the elderly population and can be fatal in its advanced stages. Key symptoms include impairments in attention, executive function, language, and other crucial cognitive abilities [3]. Globally, cognitive impairment affects over 15% of community-dwelling elderly individuals, with an even higher prevalence observed in nursing homes [4–6].

Currently, clinical assessments of cognitive impairment primarily rely on patient interviews and behavioral observations [7]. However, this approach is mainly suitable for patients with noticeable symptoms and has limitations in early screening of cognitive impairment in general populations undergoing routine health examinations. Consequently, there is an urgent need to develop a practical and effective assessment tool for the early identification of cognitive impairment. Previous studies have shown that in-depth analysis of the risk factors for cognitive impairment and the implementation of appropriate interventions can improve a patient's quality of life and delay the progression of cognitive impairment to dementia [8–10]. Many researchers have developed models to evaluate cognitive status, incorporating factors like age, sex, education level, neuroimaging data, and Magnetic Resonance Imaging (MRI) biomarkers as key features [11, 12]. While most studies focus on elderly populations, few assessment models include middle-aged and elderly individuals (≥ 40 years old). For example, Hu et al. used individuals older than 60 as study participants and collected relevant data to build machine-learning models for assessing cognitive impairment [13]. Similarly, Wang et al. established a machine learning model that used variables such as playing more mahjong or cards, gardening, and watching TV or listening to broadcasts as features to assess cognitive impairment in individuals over 65 years old [14]. Although the risk factors of cognitive impairment have been widely studied, they primarily focus on age, education level, place of residence, and sex [15]. However, these factors alone do not fully explain the complexity of the disease. Multiple studies have highlighted the association between specific lifestyles and cognitive impairment [16, 17]. Regular physical activity is recognized as critical for maintaining brain health and can effectively reduce the risk of cognitive impairment [18, 19]. Similarly, eating habits have received attention [20]. Studies suggest that a diet rich in fruits and vegetables is associated with a lower risk of cognitive impairment [21]. Social participation has also become a key factor, as maintaining strong social connections can provide emotional support and reduce the risk of cognitive impairment [22, 23]. These findings emphasize the critical role of lifestyle in preventing cognitive impairment.

Addressing these gaps, this study established a nomogram to assess cognitive impairment among 581 community-dwelling adults aged 40 years and older in China. The model integrates demographic, clinical, and lifestyle factors to provide an objective and convenient approach for evaluating cognitive health. It helps identify individuals at higher risk of impairment and supports early detection and intervention. These findings contribute to improving population-based screening and advancing strategies for preventing cognitive decline in middle-aged and older adults.

## Methods and materials

### Study design and population

This retrospective cross-sectional study analyzed data from 681 participants enrolled in a cognitive impairment screening cohort conducted in Guizhou, China, between October 23 and November 8, 2023, at the First Affiliated Hospital of the University of Science and Technology of China. The inclusion criteria were as follows: (1) age ≥ 40 years; (2) completion of the Montreal Cognitive Assessment (MoCA) and associated clinical measurements; (3) clear consciousness and no significant communication barriers. Exclusion criteria included: (1) missing data from laboratory tests and physical examination; (2) incomplete MoCA forms; (3) presence of a severe physical illness that prevented the completion of the survey. In total, 581 participants were finally enrolled. In this study, individuals aged ≥ 40 years were included and classified as middle-aged and older adults, with those aged 40–59 years defined as middle-aged and those aged ≥ 60 years defined as older adults, as this age range captures the early stage at which cognitive impairment and associated vascular risk factors may begin to develop [24–26]. This study was approved by the institutional ethics committee of the First Affiliated Hospital of the University of Science and Technology of China. Given its retrospective nature, the requirement for informed consent was waived.

### Data collection

In the baseline survey, all participants completed the MoCA questionnaire and were subsequently classified as having cognitive impairment or no cognitive impairment based on their scores. Additionally, all participants underwent physical and laboratory examinations. Physical examinations included weight, abdominal circumference (AC), systolic blood pressure (SBP), and diastolic blood pressure (DBP). Laboratory tests collected indicators such as fasting blood glucose (FBG), triglycerides (TG), total cholesterol (TC), low-density lipoprotein cholesterol (LDL-C), high-density lipoprotein cholesterol (HDL-C), homocysteine (Hcy), uric acid (UA), creatinine (Cr), Apolipoprotein B (ApoB), and estimated glomerular filtration rate (eGFR). Calculations for triglyceride glucose index (TyG) and body mass index (BMI) were also performed. Demographic characteristics, including sex and age, were also included.

### Assessment of cognitive impairment

Cognitive impairment refers to difficulties in domains encompassing memory, language, attention, reasoning, planning, or problem-solving that impair daily functioning and independence [27]. The MoCA was used for evaluation, as it is a well-validated, sensitive tool for screening subtle cognitive deficits [28, 29]. The MoCA covers multiple cognitive domains, including visuospatial/executive function, naming, attention, language, abstraction, delayed recall, and orientation, with a maximum score of 30. Given that the study population consisted primarily of middle-aged and older adults, and based on prior research in Chinese elderly populations, a cutoff score of 24 was applied to categorize participants into cognitive impairment (< 24) and no cognitive impairment (≥ 24). Several high-quality studies support a cutoff at or around 24. For example, a systematic review and meta-analysis reported that MoCA cutoffs of < 24 maximize the sum of sensitivity and specificity for detecting cognitive impairment [30]. A 2023 meta-analysis in Chinese elderly populations found that 24/25 provides diagnostic value nearly equivalent to 25/26 [31]. In a clinical sample, a revised cutoff of 23/24 offered higher specificity than the original 25/26 recommendation [32].

### Description and definition of feature variables

Continuous variables included: Age, BMI, AC, SBP, DBP, TyG, LDL-C, HDL-C, Non-HDLC, TC, FBG, UA, Cr, Hcy, ApoB, and eGFR.

The categorical variables are as follows: Sex is defined as, “male” and “female”; Smoking history was defined as, “having a smoking history” and “no smoking history”; Alcohol history was described as, “no drinking”, “moderate drinking”, and “binge drinking”; Exercise habit is defined as, “lack of exercise” and “regular exercise”; Dietary preference is described as, “balanced”, “salty” and “mild” taste preference; Carnivorous or Vegetarian preference are described as, “balanced”, “carnivorous” and “vegetarian”; Vegetables consumption: “ ≤ 2d/w”, “3-4d/w”, “ ≥ 5d/w”; Fruit consumption: “ ≤ 2d/w”, “3-4d/w”, “ ≥ 5d/w”; Napping habits is defined as, “having napping habits” and “not having napping habits”; Staying up late is defined as, “having” and “not having”; Insomnia is defined as, “never had insomnia, “occasionally insomnia”, “Frequently insomnia”; Mental stress was described as, “normal”, “mild”, and “severe”; Frequency of getting angry was described as, “never”, “occasionally”, and “usually”; Emotions causing physical discomfort as, “having” and “not having”; Family history of stroke and hypertension was defined in three categories: “present”, “absent”, and “unknown”; Cerebrovascular history was defined as, “presence” and “absence”; History of cardiac was defined as, “presence” and “absence”; Migraine is defined as, “presence” and “absence”. Definitions of variables are provided in the Supplementary File (Variable Definitions section).

### Statistical analysis

Variables with missing values were examined before analysis. As the proportion of missing data was minimal and randomly distributed across variables, cases with incomplete information were excluded from the study rather than imputed. This approach ensured that the final dataset contained complete observations for all included variables.

Continuous variables were tested for normality using the Kolmogorov–Smirnov Test. Normally distributed variables were analyzed using t-tests and expressed as mean ± standard deviation (SD). Non-normally distributed variables were analyzed using the Mann–Whitney U Test and expressed as median (interquartile range). Categorical variables were analyzed using chi-square or Fisher’s Exact Tests and expressed as frequencies (percentages).

Descriptive and inferential statistical analyses, including binary logistic regression analysis, were performed using SPSS version 27.0. Nomogram construction, receiver operating characteristic (ROC) curve analysis, and model performance evaluation were conducted using R software (version 4.2.0). A two-sided *p*-value ≤ 0.05 was considered statistically significant.

### Subgroup analysis

Subgroup analyses were conducted according to age, dividing participants into a 40–60 years group and an older group (≥ 60 years). Within each subgroup, LASSO regression was first applied to select the most relevant features associated with cognitive impairment from the full set of candidate variables. The features identified by LASSO were then entered into a binary logistic regression model to determine the independent variables within each subgroup. The discriminative ability of the resulting models was assessed by ROC curve analysis, and the area under the curve (AUC) was calculated to evaluate model performance. For visual comparison, ROC curves for both age groups were presented in a single figure. This approach enabled the identification of distinct risk profiles in each age group and the assessment of subgroup-specific model performance.

### Feature selection

The dataset was randomly divided into a training cohort (80%) and a validation cohort (20%).

To reduce multicollinearity and avoid model overfitting, Least Absolute Shrinkage and Selection Operator (LASSO) regression was performed using the “glmnet” R package. This method introduces an L1 regularization penalty to shrink less important feature coefficients toward zero, thereby achieving both feature selection and dimensionality reduction. The optimal penalty parameter (λ = 0.021) was determined through tenfold cross-validation based on the minimum mean cross-validated error. Variables with non-zero coefficients were retained for further analysis.

### Model construction

Selected features were entered into a binary logistic regression model to identify independent factors associated with cognitive impairment. Significant features (p < 0.05) in the multivariate analysis were incorporated into a nomogram using the “rms” R package to visually quantify individual risk.

### Model validation and performance evaluation

Model discrimination was assessed using the area under the receiver operating characteristic curve (AUROC). Calibration was evaluated with calibration plots and the Hosmer–Lemeshow goodness-of-fit test. Internal validation was performed using bootstrap resampling with 1,000 repetitions to estimate model stability. Clinical utility was examined using decision curve analysis (DCA) to quantify the net benefit across a range of threshold probabilities.

## Results

### Participant characteristics

A total of 581 participants were included in this study, comprising 161 males (27.7%) and 420 females (72.3%). The overall prevalence of cognitive impairment was 37.3% (217 participants). The data were randomly divided into a training cohort (*n =* 465) and a validation cohort (*n =* 116). The prevalence of cognitive impairment was 38.5% (179/465) in the training cohort and 32.8% (38/116) in the validation cohort. As presented in Table 1, there were no significant differences in the baseline characteristics between the two cohorts (all *p* > 0.05). In addition, among participants aged 40–60 years (*n =* 288), 57 individuals were identified as having cognitive impairment based on the screening criteria. In the group aged ≥ 60 years (*n =* 293), 160 individuals were identified as having cognitive impairment.

**Table 1:** Baseline characteristics of the Training and Validation cohorts

Characteristics	Total (*n =* 581)	Validation Cohort (*n =* 116)	Training Cohort (*n =* 465)	*P* value
Sex, n (%)				0.791
Female	420(72.3%)	85(73.3%)	335(72.0%)	
Male	161(27.7%)	31(26.7%)	130(28.0%)	
Smoking history, n (%)				0.616
Yes	120(20.7%)	22(19.0%)	98(21.1%)	
No	461(79.3%)	94(81.0%)	367(78.9%)	
Alcohol history, n (%)				0.118
Moderate drinking	85(14.6%)	18(15.5%)	67(14.4%)	
No drinking	467(80.4%)	88(75.9%)	379(81.5%)	
Binge drinking	29(5.0%)	10(8.6%)	19(4.1%)	
Exercise habit, n (%)				0.872
Regularly	454(78.1%)	90(77.6%)	364(78.3%)	
Lack	127(21.9%)	26(22.4%)	101(21.7%)	
Dietary preferences, n (%)				0.310
Salt Preference	92(15.8%)	13(11.2%)	79(17.0%)	
Balance	354(60.9%)	75(64.7%)	279(60.0%)	
Mild Taste Preference	135(23.2%)	28(24.1%)	107(23.0%)	
Carnivorous or vegetarian preferences, n (%)				0.606
Carnivorous	76(13.1%)	13(11.2%)	63(13.5%)	
Balance	378(65.1%)	80(69.0%)	298(64.1%)	
vegetarian	127(21.9%)	23(19.8%)	104(22.4%)	
Vegetables consumption, n (%)				0.814
≤ 2d/W	21(3.6%)	5(4.3%)	16(3.4%)	
3-4d/W	155(26.7%)	32(27.6%)	123(26.5%)	
≥ 5d/W	405(69.7%)	79(68.1%)	326(70.1%)	
Fruit consumption, n (%)				0.172
≤ 2d/W	262(45.1%)	45(38.8%)	217(46.7%)	
3-4d/W	131(22.5%)	33(28.4%)	98(21.1%)	
≥ 5d/W	188(32.4%)	38(32.8%)	150(32.3%)	
Napping habits, n (%)				0.785
Yes	219(37.7%)	45(38.8%)	174(37.4%)	
No	362(62.3%)	71(61.2%)	291(62.6%)	
Staying up late, n (%)				0.500
Yes	60(10.3%)	10(8.6%)	50(10.8%)	
No	521(89.7%)	106(91.4%)	415(89.2%)	
Insomnia, n (%)				0.189
Never	213(36.7%)	51(44.0%)	162(34.8%)	
Occasionally	256(44.1%)	45(38.8%)	211(45.4%)	
Frequently	112(19.3%)	20(17.2%)	92(19.8%)	
Mental stress, n (%)	112(19.3%)	20(17.2%)	92(19.8%)	0.923
Normal	368(63.3%)	75(64.7%)	293(63.0%)	
Mild	158(27.2%)	31(26.7%)	127(27.3%)	
Severe	55(9.5%)	10(8.6%)	45(9.7%)	
Frequency of getting angry, n (%)				0.923
Never	310(53.4%)	61(52.6%)	249(53.5%)	
Occasionally	226(38.9%)	45(38.8%)	181(38.9%)	
Frequently	45(7.7%)	10(8.6%)	35(7.5%)	
Emotions causing physical discomfort, n (%)				0.444
Yes	87(15.0%)	20(17.2%)	67(14.4%)	
No	494(85.0%)	96(82.8%)	398(85.6%)	
Family history of stroke, n (%)				0.964
No	459(79.0%)	92(79.3%)	367(78.9%)	
Yes	38(6.5%)	8(6.9%)	30(6.5%)	
Unknown	84(14.5%)	16(13.8%)	68(14.6%)	
Family history of hypertension, n (%)				0.567
No	366(63.0%)	70(60.3%)	296(63.7%)	
Yes	129(22.2%)	30(25.9%)	99(21.3%)	
Unknown	86(14.8%)	16(13.8%)	70(15.1%)	
Cerebrovascular history, n (%)				0.244
No	496(85.4%)	103(88.8%)	393(84.5%)	
Yes	85(14.6%)	13(11.2%)	72(15.5%)	
History of cardiac, n (%)				0.109
No	555(95.5%)	114(98.3%)	441(94.8%)	
Yes	26(4.5%)	2(1.7%)	24(5.2%)	
Migraine, n (%)				0.945
No	359(61.8%)	72(62.1%)	287(61.7%)	
Yes	222(38.2%)	44(37.9%)	178(38.3%)	
Age, years (median, IQR)	61.00(55.00,69.00)	60.00(55.00,68.00)	61.00(55.00,69.00)	0.442
BMI, kg/m^2^ (median, IQR)	25.20(23.01,27.77)	25.27(23.17,27.66)	25.20(22.97,27.77)	0.976
AC, cm (median, IQR)	90.00(84.00,98.00)	90.00(82.25,99.00)	90.00(84.00,97.00)	0.950
SBP, mmHg (median, IQR)	146.00(130.00,164.00)	147.50(128.00,164.75)	145.00(131.00,164.00)	0.944
DBP, mmHg (median, IQR)	84.00(77.00,93.00)	83.00(74.00,93.75)	85.00(77.00,93.00)	0.122
TyG (median, IQR)	8.76(8.38,9.19)	8.76(8.47,9.27)	8.76(8.32,9.17)	0.136
LDL-C, mmol/L (median, IQR)	3.16(2.54,3.74)	3.12(2.51,3.59)	3.17(2.55,3.79)	0.542
HDL-C, mmol/L (median, IQR)	1.41(1.19,1.65)	1.35(1.15,1.67)	1.41(1.19,1.65)	0.494
Non-HDLC, mmol/L (media, IQR)	3.62(3.01,4.24)	3.72(3.14,4.22)	3.57(3.00,4.25)	0.482
TC, mmol/L (median, IQR)	5.10(4.48,5.69)	5.10(4.62,5.61)	5.10(4.43,5.71)	0.846
FBG, mmol/L (median, IQR)	4.81(4.42,5.40)	4.80(4.42,5.40)	4.82(4.42,5.36)	0.781
UA, μmol/L (median, IQR)	321.00(269.00,392.00)	329.50(265.00,420.75)	321.00(270.00,386.50)	0.375
Cr, μmol/L (median, IQR)	63.00(54.00,75.00)	62.50(56.00,73.00)	63.00(53.00,75.00)	0.471
Hcy, μmol/L (median, IQR)	17.26(13.61,23.29)	15.99(13.38,21.15)	17.59(13.63,23.60)	0.142
ApoB, g/L (median, IQR)	0.88(0.77,1.04)	0.88(0.78,0.99)	0.88(0.76,1.05)	0.667
eGFR, μmol/L (median, IQR)	127.73(102.58,157.19)	127.78(103.92,148.89)	127.73(101.74,158.18)	0.542
Cognitive impairment, n (%)				0.253
No	364(62.7%)	78(67.2%)	286(61.5%)	
Yes	217(37.3%)	38(32.8%)	179(38.5%)	

*Abbreviations*: *BMI* Body mass index, Cr Creatinine, *AC* Abdominal circumference, *DBP* Diastolic blood pressure, *eGFR* estimated glomerular filtration rate, *FBG* Fasting blood glucose, *Hcy* Homocysteine, *HDL-C* high-density lipoprotein cholesterol, *LDL-C* Low-density lipoprotein cholesterol, *Non-HDLC* Non-high-density lipoprotein cholesterol, *SBP* Systolic blood pressure, *TC* Total cholesterol, *TyG* Triglyceride-glucose index, *UA* Uric acid, *ApoB* Apolipoprotein B

### Variable selection

**Fig. 1:**
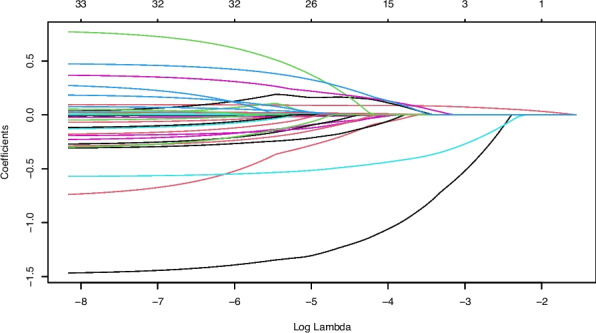
LASSO coefficient path diagram of 35 feature variables

**Fig. 2:**
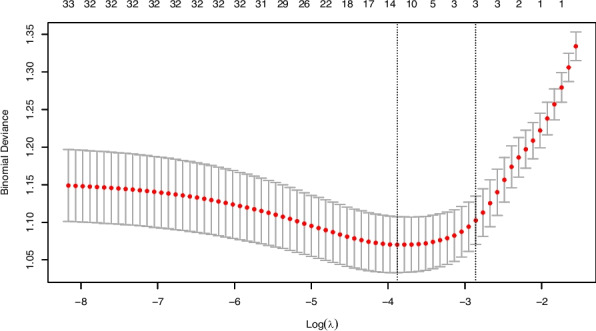
LASSO regression cross-validation plot. The two vertical dashed lines correspond to lambda. min (left) and lambda.1se (right). Based on lambda. min, 13 feature variables were selected for model construction

This study included 35 feature variables, comprising demographic information, medical history, and laboratory indicators. Using LASSO regression in R software, 13 variables were selected, as shown in Figs. 1 and 2. Figure 1 is the LASSO coefficient path plot. Figure 2 shows the cross-validation error curve. The 13 selected variables were sex, age, BMI, SBP, DBP, TyG, LDL-C, Hcy, smoking history, fruit consumption, staying up late, family history of stroke, and migraine.

### Multivariable analysis

The 13 feature variables selected by LASSO were included in a binary logistic regression analysis, which identified 6 independent variables (sex, age, SBP, Hcy, fruit consumption, and family history of stroke). Detailed information is provided in Table 2, with all variables showing a p-value less than 0.05, indicating statistically significant differences.

**Table 2:** Binary logistic regression analysis

**Variable**		**B**	***P***	**OR**	**95%CI**
Age		0.085	< 0.001	1.089	1.058–1.122
SBP		0.016	0.023	1.016	1.002–1.030
Hcy		0.023	0.010	1.023	1.005–1.041
Sex	Female	Reference			
	Male	−1.526	< 0.001	0.215	0.102–0.455
Fruit consumption	≤ 2d/W	Reference	< 0.001		
	3-4d/W	−0.322	0.286	0.724	0.400–1.310
	≥ 5d/W	−1.106	< 0.001	0.331	0.190–0.574
Family history of stroke	NO	Reference	0.084		
	YES	−0.503	0.393	0.605	0.191–1.918
	Unknown	0.644	0.048	1.903	1.006–3.599

All variables were included in the analysis as categorical features. Although some subcategories did not reach statistical significance, the significance of individual levels is reported for descriptive purposes

*Abbreviations*: *Hcy* Homocysteine, *SBP* Systolic blood pressure, *B* regression coefficient, *P* *P*-value

### Subgroup analysis by age

After subgroup analysis, distinct independent features were identified across different age groups, as shown in Tables 3 and 4. In the 40–60 years group, six variables were independently associated with cognitive impairment: age, SBP, HDL-C, male sex, fruit consumption (≥ 5 d/w), and migraine. Specifically, Age was positively associated with cognitive impairment (OR 1.143, 95% CI 1.049–1.245, *p =* 0.002), indicating that even within this middle-aged population, increasing age contributes to higher risk. SBP was a risk factor (OR 1.030, 95% CI 1.010–1.050, *p =* 0.003), suggesting that elevated systolic blood pressure may impair cognitive function. HDL-C showed a protective effect (OR 0.167, 95% CI 0.045–0.623, *p =* 0.008), highlighting the potential benefit of higher “good” cholesterol levels. Male sex was associated with increased risk (OR 0.129 for female reference, 95% CI 0.032–0.528, *p =* 0.004). Fruit consumption (≥ 5 d/w) was protective (OR 0.227, 95% CI 0.090–0.575, *p =* 0.002), whereas intermediate consumption (3–4 d/w) was not significant. History of migraine was a significant risk factor (OR 2.873, 95% CI 1.281–6.441, *p =* 0.010), suggesting that migraine may contribute to cognitive decline in middle-aged adults. The model showed good discriminative ability with an AUC of 0.812, indicating that the selected features reliably distinguish individuals with cognitive impairment in this age group.

**Table 3:** Independent variables associated with cognitive impairment in the 40–60 years subgroup (*n =* 288) identified by binary logistic regression

**Variable**		**B**	***P***	**OR**	**95%CI**
Age		0.133	0.002	1.143	1.049–1.245
SBP		0.030	0.003	1.030	1.010–1.050
HDL-C		−1.788	0.008	0.167	0.045–0.623
Sex	Female	Reference			
	Male	−2.045	0.004	0.129	0.032–0.528
Fruit consumption	≤ 2d/W	Reference	0.005		
	3-4d/W	−0.186	0.698	0.830	0.324–2.126
	≥ 5d/W	−1.483	0.002	0.227	0.090–0.575
Migraine	NO	Reference			
	YES	1.055	0.010	2.873	1.281–6.441

All variables were included in the analysis as categorical features. Although some subcategories did not reach statistical significance, the significance of individual levels is reported for descriptive purposes

*Abbreviations*: *HDL-C* high-density lipoprotein cholesterol, *SBP* Systolic blood pressure, *B* regression coefficient, *P* *P*-value

**Table 4:** Independent variables associated with cognitive impairment in the ≥ 60 years subgroup (*n =* 293) identified by binary logistic regression

**Variable**		**B**	***P***	**OR**	**95%CI**
Age		0.095	< 0.001	1.100	1.043–1.161
DBP		−0.021	0.029	0.979	0.960–0.998
TyG		0.607	0.037	1.835	1.039–3.240
Sex	Female	Reference			
	Male	0.803	< 0.013	2.323	1.186–4.200
Fruit consumption	≤ 2d/W	Reference	0.023		
	3-4d/W	−0.225	0.543	0.798	0.386–1.650
	≥ 5d/W	−0.969	0.006	0.379	0.190–0.757
Carnivorous or vegetarian preferences	Balance	Reference	0.004		
	vegetarian	1.053	0.002	2.865	1.456–5.639
	Carnivorous	−0.340	0.444	0.712	0.298–1.698

All variables were included in the analysis as categorical features. Although some subcategories did not reach statistical significance, the significance of individual levels is reported for descriptive purposes

*Abbreviations*: *TyG* Triglyceride-glucose index, *DBP* Diastolic blood pressure, *B* regression coefficient, *P* *P*-value

In the ≥ 60 years group, six independent risk factors were identified: age, DBP, TyG, male sex, fruit consumption (≥ 5 d/w), and vegetarian dietary preference. Specifically, Age remained a strong risk factor (OR 1.100, 95% CI 1.043–1.161, p < 0.001). DBP was inversely associated with risk (OR 0.979, 95% CI 0.960–0.998, *p =* 0.029), suggesting that lower diastolic pressure may be linked to cognitive impairment in older adults. TyG, a marker of insulin resistance, was positively associated with risk (OR 1.835, 95% CI 1.039–3.240, *p =* 0.037). Male sex was again a risk factor (OR 2.323, 95% CI 1.186–4.200, *p =* 0.013). Frequent fruit consumption (≥ 5 days/week) was protective (OR 0.379, 95% CI 0.190–0.757, *p =* 0.006). Vegetarian dietary preference was associated with a higher risk compared to a balanced diet (OR 2.865, 95% CI 1.456–5.639, *p =* 0.002), whereas a carnivorous preference was not significant. The discriminative performance of this subgroup model was moderate, with an AUC of 0.758, indicating that the selected features reasonably assess cognitive impairment in the older population.

**Fig. 3:**
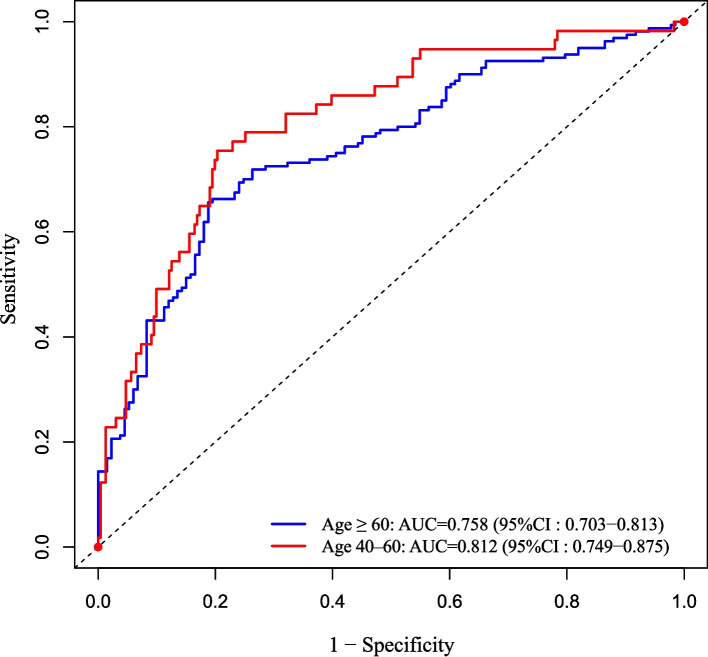
ROC curves showing discriminative performance of age-specific models for cognitive impairment. The 40–60 years subgroup (*n =* 288) is represented by the red curve (AUC = 0.812), and the ≥ 60 years subgroup (*n =* 293) is represented by the blue curve (AUC = 0.758)

Comparison between age groups: The analysis demonstrates that the 40–60 and ≥ 60 years groups have distinct risk profiles, with some overlapping (age, male sex, frequent fruit consumption) and some age-specific factors (HDL-C and migraine in 40–60; DBP, TyG, vegetarian diet in ≥ 60). These findings suggest that tailored risk assessment and prevention strategies should be considered according to age. ROC curves for both subgroups are presented in Fig. 3.

### Nomogram development

**Fig. 4:**
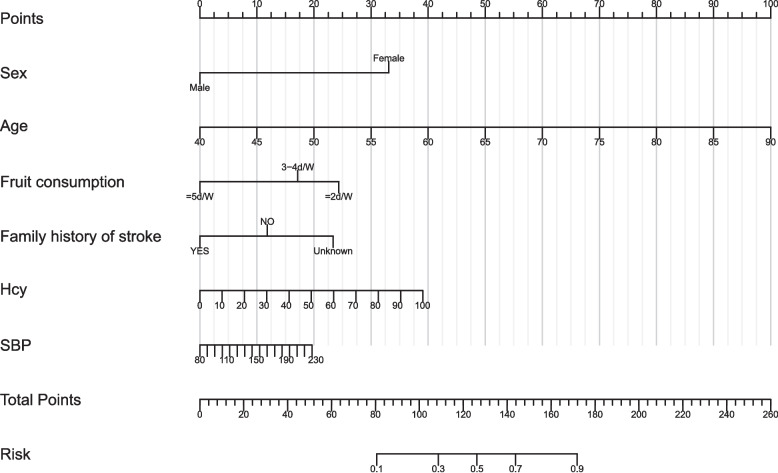
Nomogram for assessing cognitive impairment. The variables included in the nomogram were derived from binary logistic regression analysis, and each is clearly shown to contribute to the assessment of cognitive impairment. Each variable corresponds to a specific score, and the total score of the six variables yields an estimated probability of cognitive impairment. The included variables were sex, age, fruit consumption, Hcy, SBP, and Family history of stroke. Abbreviations: SBP, systolic blood pressure; Hcy, Homocysteine

Using the 6 independent variables, a nomogram was created to assess the risk of cognitive impairment, as shown in Fig. 4. The nomogram illustrates the assessment value of these six risk factors in assessing cognitive impairment. Each risk factor corresponds to a specific score, and the total score determines the probability of cognitive impairment. Higher total scores indicate a higher risk of cognitive impairment. The model’s goodness-of-fit was assessed using the Hosmer–Lemeshow test for both the training and validation cohorts, showing p-values of 0.215 and 0.369, respectively, which indicate good model fit and generalizability.

### Nomogram model evaluation

**Fig. 5:**
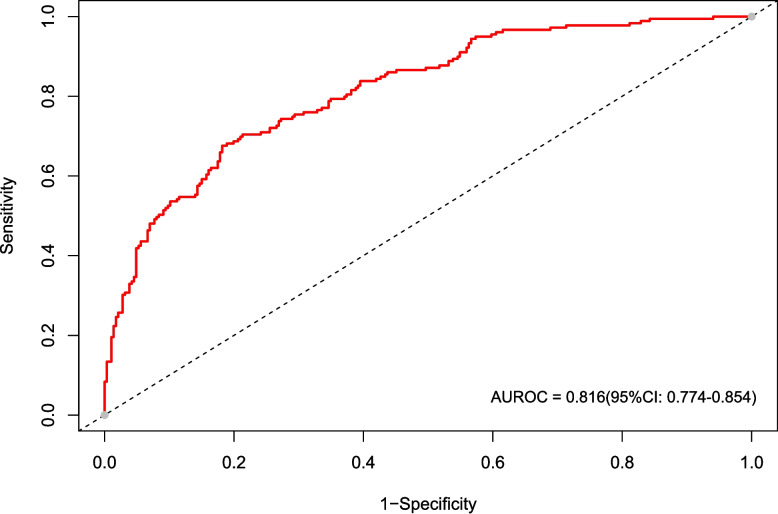
ROC curve of the training cohort. Abbreviations: AUROC, Area Under the Receiver Operating Characteristic curve

**Fig. 6:**
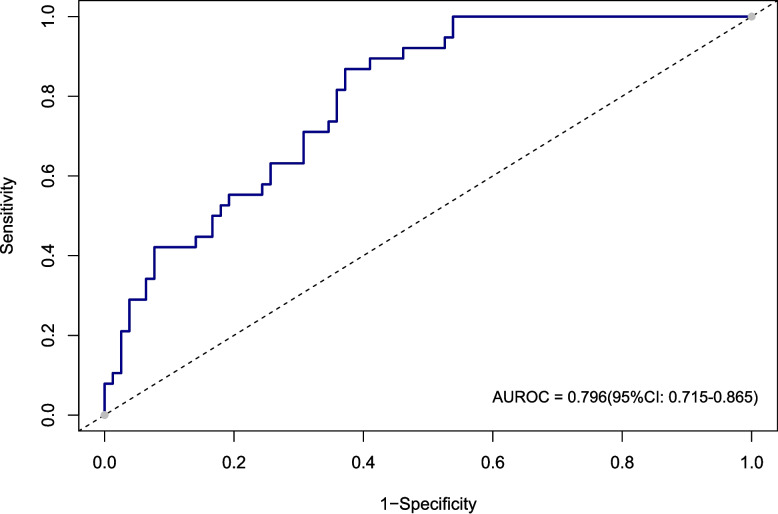
ROC curve of the validation cohort. Abbreviations: AUROC, Area Under the Receiver Operating Characteristic curve

**Fig. 7:**
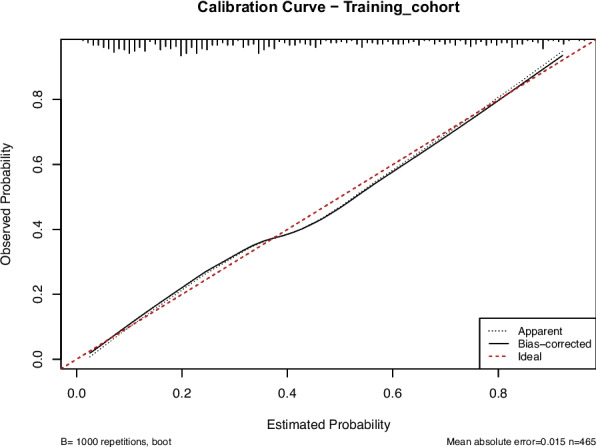
Calibration curve of training cohort

**Fig. 8:**
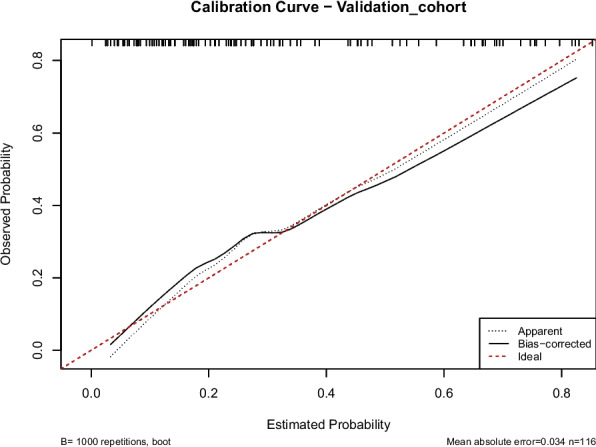
Calibration curve of validation cohort

**Fig. 9:**
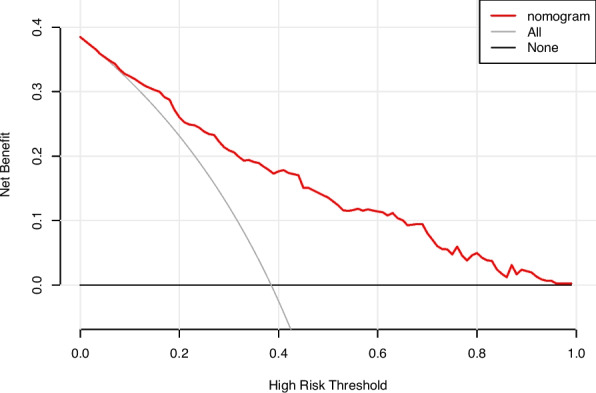
Decision curve analysis of the training cohort

**Fig. 10:**
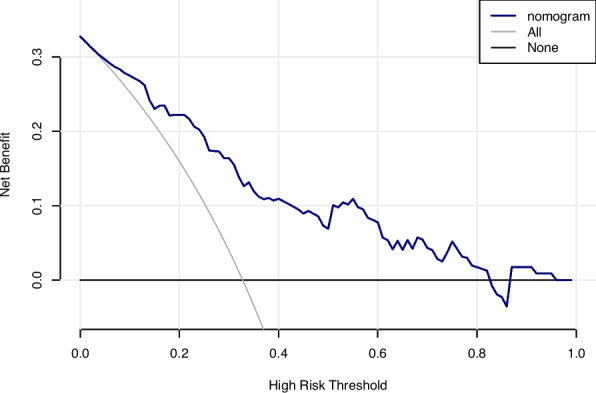
Decision curve analysis of the validation cohort

In this study, we evaluated the model's discriminative ability in the training and validation cohorts using the AUC to assess the accuracy of cognitive impairment. ROC analysis revealed AUC values of 0.816 (95% CI: 0.774–0.854) for the training cohort and 0.796 (95% CI: 0.715–0.865) for the validation cohort. These results indicate that the model has good stability and discriminative ability, as shown in Figs. 5, 6, 7 and 8 show the calibration curves for both cohorts, which compare the estimated probabilities with the actual outcomes. In the calibration curve, the dashed line labeled “Apparent” represents the model’s performance on the original dataset, whereas the solid line labeled “Bias-corrected” reflects the performance after bias correction. Ideally, the estimated probabilities should align closely with the 45° diagonal line, indicating strong agreement between estimated and observed outcomes. As shown in the figure, both curves lie near the ideal line with only minor deviations; the bias-corrected solid line is slightly below the apparent line, suggesting that the corrected estimates better correspond to the actual observations. The notation “B = 1000 repetitions, boot” indicates that 1000 bootstrap resampling iterations were performed for bias estimation and correction. In the training cohort, the mean absolute error (MAE) was 0.015 (*n =* 465), and in the validation cohort, it was 0.034 (*n =* 116). Both MAE values were below 0.05, further demonstrating the good calibration performance of the model. Additionally, we generated DCA curves for the nomogram, treatment strategies, and no-treatment strategies, shown in Figs. 9 and 10. In these plots, the x-axis represents the threshold probability for model assessment, and the y-axis represents the net benefit. A higher net benefit curve indicates that the model provides greater clinical benefit at the corresponding threshold probability. Our DCA results show that the net benefit of the model is higher than the net clinical benefit of extreme scenarios, suggesting that the nomogram model provides substantial benefit in clinical decision-making. Therefore, the nomogram developed in this study demonstrates practical utility in assessing cognitive impairment.

## Discussion

In this study, we developed a nomogram to assess the risk of cognitive impairment among middle-aged and older adults in community settings. The model integrates six readily obtainable demographic, clinical, and lifestyle variables, including age, sex, SBP, fruit consumption, family history of stroke, and Hcy levels, and demonstrates good discrimination and calibration. Notably, fruit consumption emerged as an independent protective factor alongside established variables such as age and hypertension. Importantly, there was no significant difference in cognitive impairment prevalence between the training and validation cohorts, indicating comparability in key characteristics and supporting the internal consistency of the model. This comparability also suggests that the model performs consistently across similar populations, reinforcing confidence in its internal validation and applicability in community-based screening. While both cohorts were drawn from similar populations, further validation in independent and more diverse cohorts is warranted to assess generalizability.

Having established the model’s performance and key variables, we next considered how the nomogram could guide clinical and community practice. By identifying individuals at higher risk of cognitive impairment, the tool allows clinicians and community health practitioners to tailor follow-up intensity and monitoring strategies to each person’s risk profile. This enables high-risk individuals to receive personalized management. In this way, “personalized” or “targeted” management refers to risk-stratified cognitive monitoring and referral approaches, rather than therapeutic intervention. For example, middle-aged adults with elevated SBP, migraine, or low HDL-C may benefit from more frequent cognitive follow-up or vascular monitoring, whereas older adults with low DBP, elevated TyG index, or a predominantly vegetarian diet may require closer surveillance of cerebral perfusion, metabolic status, and nutrient intake. The nomogram thus serves as a low-cost, accessible screening and triage tool, complementing neurological assessment and enabling early identification of individuals with heightened cognitive vulnerability who may benefit from closer observation or further evaluation.

Age is a well-established risk factor for cognitive impairment. Consistent with Pinyopornpanish et al., we observed an increasing risk with advancing age, particularly in individuals aged 60–65 years, among whom the prevalence reached 47% [33]. This underscores the neurological significance of early cognitive vulnerability, as aging is accompanied by progressive neuronal and synaptic loss. Pathological changes, including β-amyloid plaque deposition and neurofibrillary tangles that are hallmarks of Alzheimer’s disease, further exacerbate early cognitive decline [34]. These observations highlight the importance of systematic cognitive monitoring in middle-aged and older adults in both clinical and community settings. Age-stratified subgroup analyses further clarified these findings, revealing that the factors contributing to cognitive impairment differ between midlife and later life. In the 40–60 years subgroup, age remained an independent risk factor for cognitive impairment, indicating that even during midlife, increasing age is associated with a measurable rise in cognitive risk. In this group, higher SBP, lower HDL-C levels, and migraine were identified as important risk factors, suggesting that early vascular stress and cerebrovascular dysfunction contribute to cognitive decline. Hypertension has been shown to disrupt cerebral microvascular integrity and promote white matter damage associated with cognitive impairment [35].Migraine have also been linked to structural and functional brain changes that may impact cognition, potentially through microvascular and network alterations [36].Similarly, lower HDL-C can reduce endothelial protection and has been implicated in cognitive outcomes in older adults [37]. In contrast, in the ≥ 60 years subgroup, age exerted a stronger and more consistent effect, reflecting cumulative neurodegenerative and cerebrovascular burden in later life. In this group, lower DBP and elevated TyG were independently associated with cognitive impairment. Lower DBP may reduce cerebral perfusion, compromising oxygen and nutrient delivery to vulnerable brain regions, contributing to neuronal injury and cognitive deterioration [38]. Elevated TyG index, a surrogate marker of insulin resistance, has been associated with increased risk of cognitive impairment and dementia, highlighting the role of metabolic dysregulation and its vascular consequences in late-life cognitive decline [39]. Notably, dietary patterns showed age-specific associations with cognition. While frequent fruit consumption remained protective across age groups, a predominantly vegetarian dietary preference in older adults was linked to a higher risk of cognitive impairment [40]. Although plant-based diets are often considered cardioprotective, in this context, the association may reflect insufficient intake of key neuroprotective nutrients, such as vitamin B12, omega-3 fatty acids, and high-quality protein, which are essential for maintaining neuronal integrity and cerebrovascular function in later life [41, 42]. Based on these findings, older adults may benefit from ensuring adequate intake of fruits, high-quality protein, and vitamin B12 to support brain health and cognitive resilience. Sex differences also highlight how cognitive risk changes with age. Sex also emerged as a significant factor, with cognitive impairment more prevalent in women (41.7%) than in men (26.1%) [43, 44]. In our cohort, cognitive impairment was overall more common in women than in men. Subgroup analyses showed that in adults aged 40–60 years, women had a higher risk of cognitive impairment, whereas in adults aged 60 years and older, men were at greater risk. These observations suggest that midlife cognitive vulnerability in women may be influenced by hormonal fluctuations, metabolic differences, and vascular factors, consistent with evidence from observational studies [45, 46]. Meanwhile, in older men, accumulated vascular and metabolic burden may increase susceptibility to cognitive decline, reflecting sex-specific patterns of vascular contributions to cognitive impairment across the lifespan [47]. Although these mechanisms are plausible, they remain speculative and warrant further investigation. Lifestyle factors, particularly fruit consumption, were consistently associated with cognitive outcomes across age groups. Participants consuming fruit ≥ 5 days per week exhibited a lower risk of cognitive impairment, likely reflecting the neuroprotective effects of antioxidants, vitamins, and polyphenols in reducing oxidative stress and neuroinflammation [48, 49]. Regular fruit intake may help preserve neuronal integrity, maintain synaptic function, and slow age-related cognitive decline, thereby lowering the likelihood of developing mild cognitive impairment or other forms of cognitive dysfunction [50]. Conversely, inadequate fruit consumption may fail to counteract oxidative and inflammatory processes, increasing susceptibility to cognitive deficits over time [51, 52]. Regarding family history of stroke, our overall analysis indicated that it was not significantly associated with cognitive impairment, and this variable did not enter any age-stratified subgroup models. Interestingly, participants reporting an unknown family history exhibited a higher risk of cognitive impairment, which may reflect differences in health awareness, access to medical information, or engagement in preventive care rather than a direct biological mechanism. In both clinical practice and community health settings, assessing family history can help identify individuals who may benefit from enhanced cognitive monitoring and educational support. Lastly, elevated Hcy levels were confirmed as an important risk factor. Increased plasma Hcy may accelerate cognitive decline through mechanisms such as oxidative stress, neuronal damage, and endothelial dysfunction, thereby contributing to cerebrovascular lesions and impaired cerebral blood supply [53–58]. Although homocysteine was identified as a significant risk factor in the overall population, it was not consistently retained in the final models of the subgroup analyses. This is because the purpose of subgroup analyses was to explore population-specific risk profiles rather than to replicate the overall model. Consequently, some variables that were significant in the full cohort, including homocysteine, did not remain independently associated with cognitive impairment across all subgroups. This observation underscores the heterogeneity of risk factor patterns for cognitive impairment and highlights the importance of tailored risk assessment in different demographic groups.

From a clinical and community perspective, the nomogram does not prescribe intervention but informs how cognitive monitoring and referral intensity may be adjusted according to an individual’s risk profile. For example, middle-aged adults with elevated SBP, migraine, or low HDL-C may benefit from more frequent cognitive follow-up, whereas older adults with low DBP, elevated TyG, or a predominantly vegetarian diet may benefit from closer surveillance of cerebral perfusion, metabolic status, and nutrient intake. Although the cross-sectional design precludes direct inference regarding progression to dementia, early recognition of these vascular, metabolic, and lifestyle risk patterns may indirectly mitigate progression by enabling timely evaluation before irreversible neurodegenerative changes occur.

Taken together, these findings place the nomogram within a neurological framework of vascular cognitive impairment and early cognitive vulnerability. As a pragmatic, community-based screening and triage tool, it bridges population-level risk identification and specialized neurological evaluation, guiding subsequent assessment or referral in a resource-efficient manner rather than serving as a diagnostic instrument.

### Limitations

Although the nomogram developed in this study demonstrated good performance in assessing cognitive impairment, several limitations should be noted. First, this was a single-center, cross-sectional study with participants recruited from rural primary care settings, limiting generalizability and precluding causal inference, and the model was not validated in an independent external cohort. While internal validation using bootstrap resampling indicated stability, external validity remains uncertain, and overfitting cannot be ruled out; therefore, the nomogram should be considered an exploratory risk stratification tool. Second, some variables, including mental stress, frequency of anger, and emotion-related physical discomfort, were assessed via self-report without standardized instruments, which may introduce misclassification or bias. Third, education level, a critical determinant of cognitive screening performance, was not available, potentially affecting the specificity of the nomogram and leading to overestimation of risk in populations with lower educational attainment. Future multicenter, larger, and more diverse longitudinal studies are warranted to validate and optimize the nomogram and enhance its utility in clinical and community-based risk assessment. 

## Conclusion

In conclusion, the developed nomogram provides an exploratory tool for identifying middle-aged and older adults at high risk of cognitive impairment. Early identification allows risk-stratified follow-up and targeted preventive measures, including blood pressure control, regular physical activity, adherence to a balanced diet rich in fruits and vegetables, and engagement in cognitively stimulating activities. These strategies are supported by evidence linking vascular risk management, healthy diet, exercise, and cognitive engagement to reduced risk of cognitive decline. By guiding risk-stratified management and optimizing healthcare resources, this tool may help slow cognitive deterioration and improve overall cognitive health. Future external and multicenter validation is warranted to confirm its generalizability and clinical utility.

## Supplementary Information

Supplementary Material 1.

## Data Availability

The data that support the findings of this study are available from the corresponding author, upon reasonable request.

## Abbreviations

AD: Alzheimer’s Disease
MoCA: Montreal Cognitive Assessment
ROC: Receiver Operating Characteristic
DCA: Decision Curve Analysis
MRI: Magnetic Resonance Imaging
AC: Abdominal circumference
SBP: Systolic blood pressure
DBP: Diastolic blood pressure
FBG: Fasting blood glucose
TG: Triglycerides
TC: Total cholesterol
LDL-C: Low-density lipoprotein cholesterol
HDL-C: High-density lipoprotein cholesterol
Hcy: Homocysteine
UA: Uric acid
Cr: Creatinine
ApoB: Apolipoprotein B
eGFR: Estimated glomerular filtration rate
TyG: Triglyceride glucose index
BMI: Body mass index
LASSO: Least Absolute Shrinkage and Selection Operator
MAE: Mean absolute error
