# Supplementary material for: Development of a clinical nomogram to assess the risk of cognitive impairment in community-dwelling middle-aged and older adults
Source: BMC Neurol. 2026 Feb 11;26:171. doi: 10.1186/s12883-026-04719-6 (PMC12997881; doi:10.1186/s12883-026-04719-6)
Supplement: Supplementary file 1 — Supplementary Material 1. [file 12883_2026_4719_MOESM1_ESM.docx]

Development of a Clinical Nomogram to Assess the Risk of Cognitive Impairment in Community-Dwelling Middle-Aged and Older Adults

1. **Variable Definitions**

We used a questionnaire-based assessment, in which some of the variables were defined as follows:

**Moderate drinking is defined as consuming no more than 1 standard drink per day for women and 2 standard drinks per day for men, where 1 standard drink contains approximately 10–14 g of pure alcohol, consistent with WHO guidelines and prior epidemiological studies. Binge drinking was defined as consuming 4 or more standard drinks for women or 5 or more standard drinks for men on a single occasion**[1]**.**

**Exercise habit** is defined as “lack of exercise” and “regular exercise.” Regular exercise was defined as engaging in moderate-intensity physical activity for at least 30 minutes per session on no fewer than three days per week, based on self-reported information. This definition was adopted in accordance with established international physical activity guidelines[2].

**Dietary preference** is described as “balanced”, “salty”, and “mild” taste preference. In this study, we focused on participants classified as having a mild taste preference, who reported consuming relatively small amounts of salt or preferring less salty foods in their usual diet. This operational definition is consistent with prior epidemiological evidence demonstrating that self-reported salt taste preference is associated with actual sodium intake[3].

**Fruits and vegetables intake** is assessed based on self-reported usual consumption and quantified as the total intake of all types of fruits and vegetables, without distinguishing individual varieties. Responses were converted into standardized portions per day. This operational definition follows prior epidemiological studies that used total fruit and vegetable intake as a meaningful indicator of overall dietary quality[4] and as a key component of dietary patterns associated with cognitive outcomes[5].

**Staying up late** is defined as “having” and “not having”. Staying up late was assessed based on self-reported habitual bedtime. Participants who reported going to bed later than 23:00 on most days were classified as having a “staying up late” habit. This operational definition was informed by prior epidemiological studies that grouped participants based on bedtime categories including >23:00, which has been associated with adverse health outcomes such as cardiovascular events and altered hypertension risk in large cohort analyses[6,7].

**Insomnia** is defined as, “never had insomnia”, “occasionally insomnia”, “Frequently insomnia”, Insomnia was assessed using a self-reported questionnaire and categorized based on frequency as follows: “Never” (participants who did not experience insomnia), “Occasionally” (<1 time per week), and “Frequently” (2–3 times per week)

**Mental stress** is categorized into three levels—normal, mild, and severe—based on participants' self-reported symptoms, daily functioning, and subjective experience. While no formal psychometric scale was used, these categories were determined according to commonly accepted clinical judgment criteria.

**The frequency of anger** is determined based on participants' self-reported experiences and observed behaviors, categorized as low, moderate, or high. No formal psychometric instrument was used.

**Physical discomfort caused by emotions** is defined as somatic symptoms such as increased heart rate, headache, or gastrointestinal discomfort that participants attributed to emotional states. These were recorded based on self-report and clinical judgment, rather than using a validated instrument.

1. Understanding alcohol drinking patterns | national institute on alcohol abuse and alcoholism (NIAAA) [Internet]. [cited 2026 Jan 6]. Available from: https://www.niaaa.nih.gov/alcohols-effects-health/alcohol-drinking-patterns

2. CDC. Physical Activity Basics. 2025 [cited 2025 Dec 30]. Adult activity: An overview. Available from: https://www.cdc.gov/physical-activity-basics/guidelines/adults.html

3. He Q, Du X, Wang L, Fang Y, Zhong J, Hu R. Taste preference for salt predicts salt intake in a Chinese population. Nutrients. 2024 June 29;16(13):2090.

4. Boeing H, Dietrich T, Hoffmann K, Pischon T, Ferrari P, Lahmann PH, et al. Intake of fruits and vegetables and risk of cancer of the upper aero-digestive tract: The prospective EPIC-study. Cancer Causes Control. 2006 Sept;17(7):957–69.

5. Morris MC, Tangney CC, Wang Y, Sacks FM, Barnes LL, Bennett DA, et al. MIND diet slows cognitive decline with aging. Alzheimers Dement. 2015 Sept;11(9):1015–22.

6. Su Y, Ding N, Zhou Y, Yang G, Chai X. The association between bedtime at night and hypertension in adults. Postgrad Med. 2023 May;135(4):370–8.

7. Yan B, Li R, Li J, Jin X, Gao F, Gao Y, et al. Sleep timing may predict congestive heart failure: A community-based cohort study. J Am Heart Assoc. 2021 Mar 16;10(6):e018385.
